# PCP4 inhibits the progression of prostate cancer through Ca^2+^/CAMKK2/AMPK/AR pathway

**DOI:** 10.3389/fimmu.2025.1616046

**Published:** 2025-07-17

**Authors:** Wenqiao Jia, Zeyuan Yu, Feifei Sun, Ping Liu, Bo Han

**Affiliations:** ^1^ Department of Health Management Center, Qilu Hospital of Shandong University, Jinan, China; ^2^ The Key Laboratory of Experimental Teratology, Ministry of Education and Department of Pathology, School of Basic Medical Sciences, Cheeloo College of Medicine, Shandong University, Jinan, China; ^3^ Department of Pathology, Qilu Hospital of Shandong University, Jinan, China; ^4^ Department of Pathology, Peking University People’s Hospital, Peking, China

**Keywords:** PCP4, PEP-19, prostate cancer, progression, CRPC

## Abstract

**Background:**

The development of prostate cancer (PCa) remains a major health threat for men worldwide. Calcium/Calmodulin signaling pathway has been implicated to the initiation and progression of diverse human cancers. Loss or downregulation of Purkinje cell protein 4 (PCP4), is frequently observed in some prostate cancer patients, particularly those with castration-resistant prostate cancer (CRPC).

**Methods:**

Public datasets were used to analyze PCP4 expression and the relationship between PCP4 expression and clinicopathological characteristics of PCa patients. Gain- and loss-of-function studies in PCa cell lines and mouse models were performed to characterize the role of PCP4 in tumor progression. A series of molecular and biochemical experiments were carried out in PCa cell lines to investigate the mechanism underlying PCP4-mediated tumor suppression.

**Results:**

(1) *PCP4* gene loss occurs at high frequency in PCa patients, and decreased expression of PCP4 correlates with poor prognosis of PCa, particularly CRPC development; (2) *TMPRSS2-ERG* fusion frequently co-occurs with *PCP*4 deletion; (3) PCP4 suppresses prostate cancer progression *in vitro* and *in vivo*; (4) PCP4 is an androgen receptor (AR) suppressed gene; (5) PCP4 was involved in the stabilization of CAMKK2 protein; (6) PCP4 inhibits PCa progression by regulating Ca^2+^/CAMKK2/AMPK/AR signaling axis.

**Conclusion:**

Our findings elucidate the molecular mechanism that PCP4 downregulation promotes PCa progression via Ca^2+^/CAMKK2/AMPK/AR pathway, highlighting its significance in CRPC development.

## Introduction

Prostate cancer (PCa) represents the second leading cause of cancer-related mortality among men in the United States (1). Its progression typically begins with prostatic intraepithelial neoplasia (PIN), advances to hormone naïve clinically localized prostate adenocarcinoma, and ultimately evolves into androgen-independent metastatic cancer (2). Specific genetic alterations critically drive PCa development and progression. For instance, the fusion of the transmembrane serine protease 2 (*TMPRSS2*) with the erythroblast transformation-specific-related gene (*ERG*), or *TMPRSS2-ERG* gene fusion, which frequently occurs in PCa as a result of either insertion chromosomal rearrangement or intrachromosomal deletion. The latter process involves the deletion of an approximately 3-megabase (Mb) interstitial region encompassing ~21 genes, including the Purkinje cell protein 4 (*PCP4)/PEP19*. Prior research indicated that deletion of these interstitial genes between *TMPRSS2* and *ERG* accelerates PCa progression (3). Furthermore, several tumor suppressor genes within this interstitial region, such as *Ets2* (3) and *FAM3B* (4), have been identified.

PCP4 was initially identified as a 7.6-kDa polypeptide in neonatal rats (5) and is abundantly expressed in the cerebral system (6). It is a calmodulin (CaM)-binding peptide that modulates diverse pathophysiological processes, particularly within the central nervous system (7, 8). PCP4 binds with CaM and accelerates the rates of Ca^2+^ binding to the C-domain of CaM (9). Nevertheless, the exact mechanism by which PCP4 regulates the Ca^2+^/CaM signaling in PCa cell remains unclear. Recent studies have revealed that PCP4 is implicated in tumorigenesis, as well as the migration, invasion and apoptosis of breast cancer cell lines (10–12). PCP4 has also been found to impede neurite outgrowth and neuronal differentiation in human neuroblastoma cells (13). In mucoepidermoid carcinoma, PCP4 expression was associated with improved prognosis (14). Multiple studies propose PCP4 as a novel prognostic biomarker for cancers, including lung adenocarcinoma (15), PCa (16), thyroid carcinoma (17) and testicular germ cell tumors (18). Notwithstanding, to date, no research has been conducted on the specific role that PCP4 plays and the related mechanisms within the context of PCa.

Calcium/calmodulin-dependent kinase kinase 2 (CAMKK2) has been implicated in PCa progression through to the CAMKK2-AMPK signaling pathway (19). Furthermore, CAMKK2 forms a feedback loop with the androgen receptor (AR) in PCa progression (20, 21). This kinase modulates key metabolic process by directly phosphorylating AMP-activated protein kinase (AMPK) at threonine 172 (AMPK(Thr172)), thereby generating active p-AMPK.

In this study, we demonstrate that PCP4 expression is significantly decreased in castration-resistant-prostate-cancer (CRPC) compared to both primary localized PCa and normal prostate tissues. Critically, downregulation of PCP4 can promotes the PCa progression via Ca^2+^/CAMKK2/AMPK/AR signaling axis. Consequently, we identify that PCP4 represents a novel target for PCa.

## Methods

### Cell lines and culture

The human PCa cell lines (LNCaP, C4-2B, VCaP, 22Rv1, PC3, DU145) and HEK293T cell (CRL-3216) were obtained from the American Type Culture Collection (Rockville, MD). Fetal bovine serum (FBS) and Charoal-stripped fetal bovine serum (CSS, depleted androgen and any other steroids) were purchased from Hyclone (South Logan, UT, USA). Dihydrotestosterone (DHT) was acquired from Meilunbio (Dalian, China). Thapsigargin and BAPTA-AM were purchased from APExBIO (Houston, USA). STO-609 was obtained from MedChemExpress (Monmouth Junction, NJ, USA).

### Immunofluorescence

The immunofluorescence protocol followed our established methodology (22, 23). Briefly, pretreated PCa cells were plated onto glass coverslips. After being fixed, cells were incubated with primary antibodies overnight at 4°C, Subsequently, the cells were incubated with secondary antibodies. To stain the nuclei, DAPI (Invitrogen, Carlsbad, CA, USA) was employed. The images of Immunofluorescence were captured and processed using Confocal Microscope (LSM880; ZEISS, Oberkochen, Germany). The primary antibody includes PCP4 (Proteintech, Wuhan, China) and CAMKK2 (Proteintech, Wuhan, China).

### Western blotting and immunoprecipitation

Western blotting assay was described in our previously study (24). Cells were harvested and lysed in RIPA buffer supplemented with protease inhibitors followed by protein quantification performed using BCA protein assay kit (Beyotime Biotechnology, Beijing, China). Then total protein samples along with protein ladder were loaded into the wells of SDS-PAGE gels. Wet transfer systems were used to transfer the proteins to the methanol activated PVDF membrane (Merck Millipore, Billerica, MA, USA). Then, we incubated the membranes in 10% milk for more than 1 hour to block of membranes. Primary antibody for PCP4 was obtained from Abcam (Cambridge, MA, USA). Antibodies for CAMKK2 and CaM were from Proteintech (Wuhan, China). Antibodies targeting p-AMPK, AMPK, AR, GAPDH and LaminA/C were form Cell Signaling Technology (Boston, MA, USA). Immunoprecipitation assay was conducted following the manufacturer’s instructions.

### RNA isolation and quantitative real time-PCR

The total RNA was extracted from cultured cells by the TRIzol regents (Invitrogen, Carlsba d, CA, USA) to break open the cells and release the RNA. Then mRNA was reverse-transcribed into cDNA by the ReverTra Ace qPCR RT kit (TOYOBO, Japan). FastStart Universal SYBR Green Master (Roche, Base I, Switzerland) was used for qRT-PCR assay. The details of the primers of genes used in this study are listed in **Supplementary Table S1**.

### Transient transfection and lentiviral transduction

PCP4-specific siRNAs, PCP4 plasmids and corresponding negative controls for transient transfection were designed and synthesized by Geme-Pharma (Shanghai, China). The effective sequences of siRNA are presented in **Supplementary Table 1**. To minimize off-target effects, co-transfection of two RNAs with better interference efficiency was performed. PCa cell lines were transiently transfected with siRNA or plasmids, along with their matched controls using Lipofectamine 3000 (Invitrogen, Carlsbad, CA, USA). Lentiviral vectors encoding *PCP4* and the empty vector controls were procured from Genechem (Shanghai, China), with infections performed according to the manufacturer’s instruction. Then incubate the cells with puromycin for 2 weeks to obtain cell lines with stable expression.

### Cell proliferation and migration/invasion assays

3-(4,5-dimethylthiazol-2-yl)-5-(3-carboxymethoxyphenyl)-2-(4-sulfoohenyl)-2H-tetrazolium inner salt (MTS) (Promega, Madison, WI, USA) assay was applied to evaluate cell proliferation. Pretreated cells were seeded into 96-well cell plates at a density of 1.5 × 10 (3) cells per well, with 3 replicated wells per experimental group. 10 μL of MTS solution was introduced to each well and incubated at 37 °C for 2.5 hours. Then transfer the plate to a microplate reader. Measure the absorbance at a wavelength of 490 nm. The absorbance value was proportional to the number of viable cells, reflecting the cell proliferation rate. The transwell assay was used to measure the migration and invasion ability of PCa cells.

### Tumor Xenografts

Male nude mice (4-6-week-old) were purchased from Weitonglihua Biotechnology (Beijing, China) and randomly divided into the experimental groups and control groups (n = 5/group). C4-2B cells stably expressing control or PCP4 (n = 1 × 10 (7)) were collected and resuspended in 100 μL PBS (50% Matrigel) and then injected subcutaneously into the mice. Tumor volume [length (mm)*width (2) (mm)*0.5] and tumor weight (g) were measured until 24 days after implantation. The experimental protocol was approved by the Shandong University Animal Care committee (Document No. ECSBMSSDU2021-2-126). All procedures were performed in compliance with the institution’s guidelines.

### RNA sequence and Bioinformatics analysis

We performed RNA-seq analyses (ShenZhen BGI Genomics Co., Ltd. China) to compared the mRNA expression profiles between control (Vector) and PCP4 overexpression (PCP4) C4-2B cells. The raw data is available in NCBI website (https://www.ncbi.nlm.nih.gov/geo/query/acc.cgi?acc=GSE293745). Gene Ontology/Kyoto Encyclopedia of Genes and Genomes (GO/KEGG) database was used to demonstrate the related pathways of PCP4 (25–27).

Datasets including GSE35988, GES68882, GES32269, GSE26367, GSE21034, GSE70770 and GSE70769 were downloaded from GEO database (http://www.ncbi.nlm.nih.gov/geo, modified: July 16, 2024). CBioPortal (http://www.cbioportal.org/, v6.0.20) tool was applied to assess PCP4 alteration frequency, mutation type and CNA (copy number alteration) of prostate cancer samples (28–30). The Cancer Genome Atlas (TCGA, prad_tcga_pan_can_atlas_2018), the Stand Up To Cancer (SU2C, prad_su2c_2019), MSKCC (MSKCC cancer cell 2010), and prostate_dkfz_2018 datasets were downloaded from CBioPortal website.

GSEA v4.0 program was used for gene set enrichment analysis according to the instructions. Androgen-induced and -repressed gene sets were obtained from Zhang et al. (31) and the data source and lists of these gene sets were showed in **Supplementary Table 2**.

### Measurement of cytosolic Ca^2+^ concentration

Cytosolic Ca^2+^ concentration was measured with the fluorescent using Fluo-acetoxymethylester probe (Fluo-4 AM) (Beyotime, Shanghai, China) according to manufacturer’s instruction. Briefly, after treatment, pretreated cells in 6-well plates were incubated with 5 μM Fluo-4 AM at 37°C for 1 hour in the dark and then washed 3 times with PBS to remove the extracellular Fluo-4 AM. Then, cells were incubated for another 20 min to ensure that Fluo-4 AM was completely convert into Fluo-4. Ca^2+^ imaging was acquired with inverted Fluorescence Microscope (EVOS M5000) and the fluorescent intensity was detected using flow cytometry.

### Statistical Analysis

Statistical analyses were performed using SPSS 20.0 (IBM Corp., Armonk, NY, USA) or GraphPad Prism 9.3.0. Each assay *in vivo* was performed in biological triplicate and the data are presented in the form of the mean ± standard deviation (SD). Statistical comparisons between groups were carried out using two-sided Student’s *t* test. Kaplan-Meier analysis was used to compare the survival information. The statistical significance of differences among groups was indicated by asterisks (*, *p* < 0.05; **, *p* < 0.01; ***, *p* < 0.001; ****, *p* < 0.0001). *P* < 0.05 was considered statistically significant.

## Results

### PCP4 is down-regulated in CRPC

**Figure 1:**
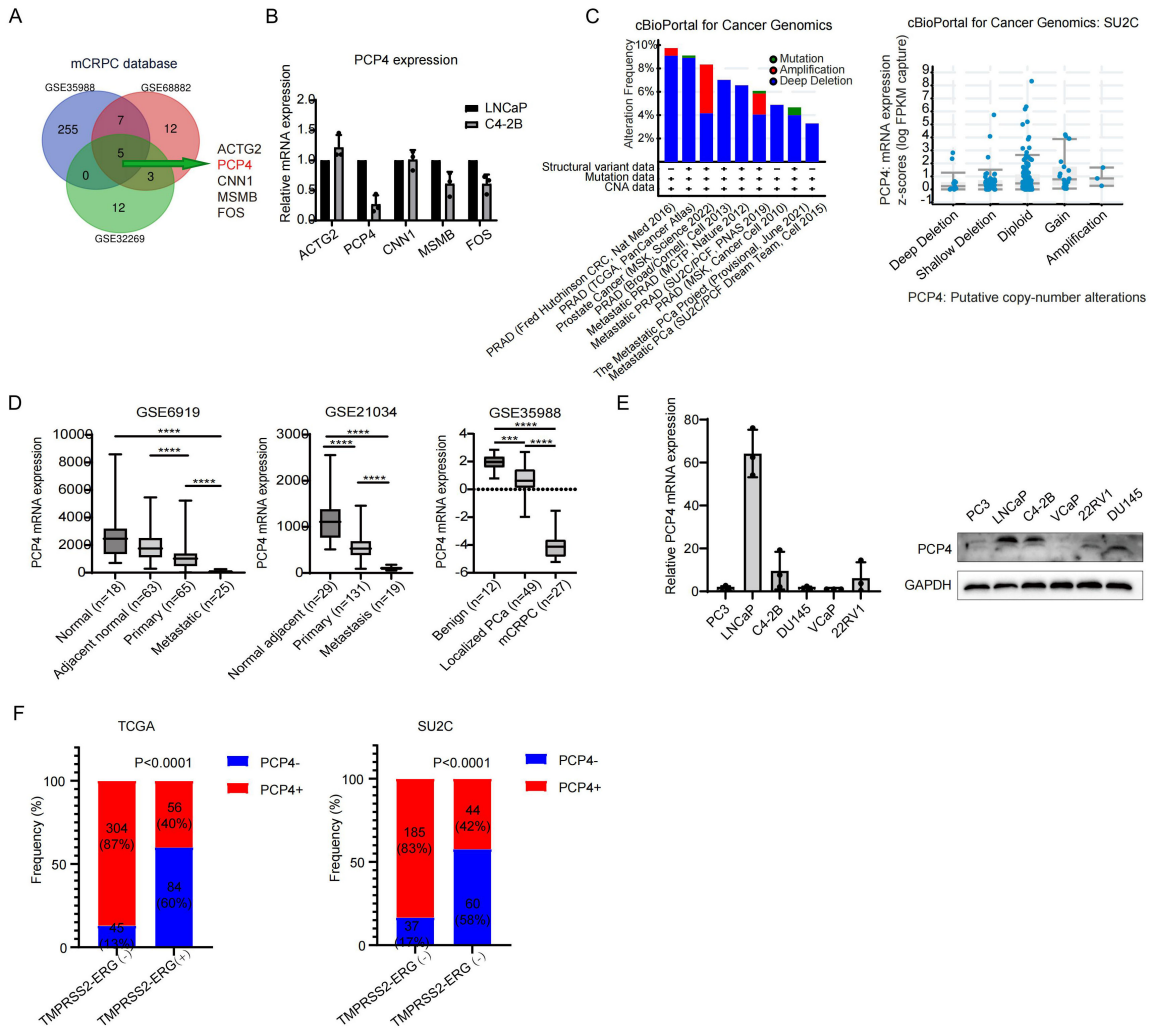
PCP4 is down-regulated in CRPC. **(A)** A Venn diagram including 3 GEO databases (GSE35988, GSE68882, GSE32269) containing primary PCa samples and CRPC samples. **(B)**. Relative transcriptional expression of the 5 key genes identified from **(A)** in PCa cell lines LNCaP and C4-2B. **(C)** The DNA alteration frequency and its relationship with transcriptomic expression of PCP4 in PCa. **(D)** The mRNA expression of PCP4 in normal adjacent prostate cancer, primary PCa and mCRPC samples. The statistical analysis was based on Student’s *t* test. **(E)** PCP4 expression in different PCa cell lines by real-time PCR and Western blotting. **(F)** Frequency of PCP4 deletion and *TMPRSS2-ERG* fusion in primary PCa and CRPC samples obtained from TCGA and SU2C datasets. mCRPC, metastatic castration resistant prostate cancer. TMPRSS2-ERG, the fusion of the transmembrane serine protease 2 with the erythroblast transformation-specific-related gene. *p<0.05, **p<0.01, ***p<0.001, ****p<0.0001.

Aiming to pinpoint the potential genes underlying the progression of PCa, particularly that of CRPC, we contrasted several datasets of primary localized PCa samples and CRPC samples, including GSE35988, GSE68882 and GSE32269. The transcriptomes between metastatic CRPC (mCRPC) and primary localized PCa samples were analyzed. By Cross-comparing all clustered genes, we obtained a common list of 5 genes that exhibited the most significantly changes in the CRPC group (> 3-fold change, *p* < 0.01; **Figure 1A**, **Supplementary Table 3**). Subsequently, the mRNA levels of these 5 genes were measured in LNCaP cells and C4-2B cells, which are a hormone sensitive prostate cancer (HSPC) model cell line and a CRPC model cell line, respectively. Among these 5 genes, PCP4 showed the most significant change, specifically a down-regulation (**Figure 1B**). A high frequency of PCP4 gene deletion was observed in different prostate adenocarcinoma studies based on cBioportal database. Moreover, the deletion of PCP4 DNA was found to be correlated with a lower transcriptome expression level of PCP4 (**Figure 1C**). Consistent with our findings, PCP4 expression was decreased in CRPC group than in primary prostate cancer tissues and normal prostate tissues in datasets such as GSE6919, GSE21034 and GES35988 (**Figure 1D**). Microarray analysis of the copy-number variation of PCP4 revealed a positive correlation between PCP4 copy-number variation and its expression (16).

PCP4 expression in different prostate cancer cell lines was performed in both transcriptome and protein level. Highest expression was observed in LNCaP cell line and lower expression lever was observed in C4-2B cell line, while extremely low expression was noted in other cell lines (**Figure 1E**).

### The relationship between PCP4 gene deletion and TMPRSS2-ERG fusion

To determine the frequency of PCP4 deletion and TMPRSS2-ERG genomic fusions in different prostate tissue types, we undertook an in-depth analysis of public datasets including TCGA and the SU2C dataset (**Figure 1F**). Regarding TCGA patients, TMPRSS2-ERG fusion was detected in 28.6% (140/489) of localized primary prostate cancer out of which 60% (84/140) exhibited both PCP4 deletion and TMPRSS2-ERG fusion. Meanwhile, concomitance of PCP4 deletion and TMPRSS2-ERG fusion was observed in CRPC patients in SU2C database.

### Down-regulation of PCP4 contributes to poor prognosis in PCa patients

**Figure 2:**
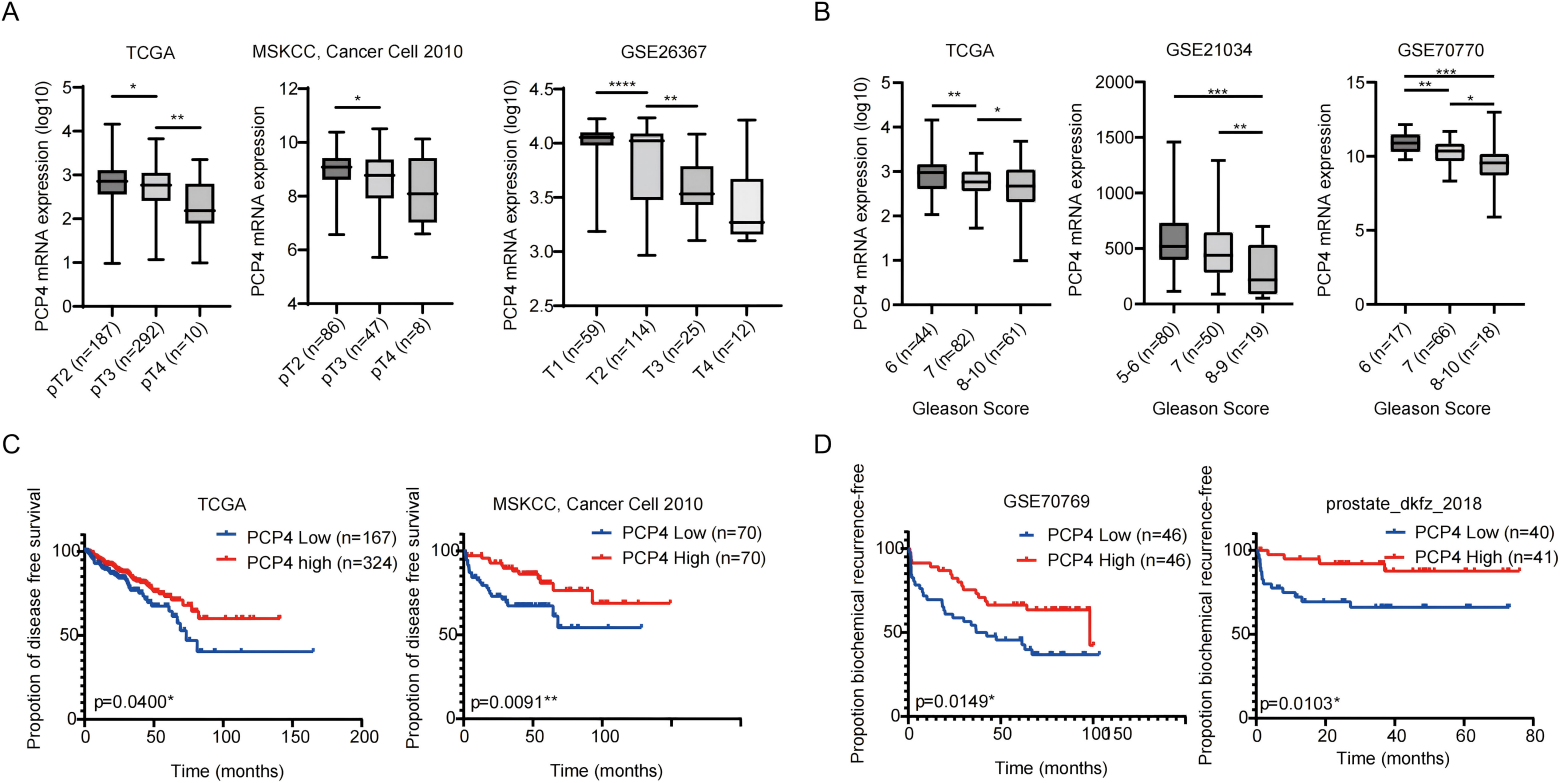
The mRNA expression characteristics of PCP4 and its relationship with PCa prognosis. **(A, B)** PCP4 expression in PCa patients with different stages and Gleason scores in public datasets (TCGA, MSKCC, GSE26367, GSE21034, GSE70770). The statistical analysis was based on Student’s *t* test. **(C)** Association between PCP4 expression and clinical outcomes. PCa cases in TCGA, MSKCC, GSE70769 and prostate_dkfz_2018 were stratified based on PCP4 expression levels and analyzed for disease-free survival or biochemical recurrence-free survival. The *p* values for Kaplan-Meier curves were determined using a log-rank test. ^*^ *p*<0.05, ^**^ *p*<0.01, ^***^ *p*<0.001, ^****^ *p*<0.0001.

TCGA, MSKCC, prostate_dkfz_2018 or GEO databases showed that reduced PCP4 mRNA expression was significantly associated with higher T stages (**Figure 2A**), higher Gleason scores (**Figure 2B**), poorer disease-free survival (DFS) (**Figure 2C**) or biochemical recurrence-free survival (BCRFS) (**Figure 2D**).

### PCP4 inhibits PCa progression *in vitro* and *in vivo*

**Figure 3:**
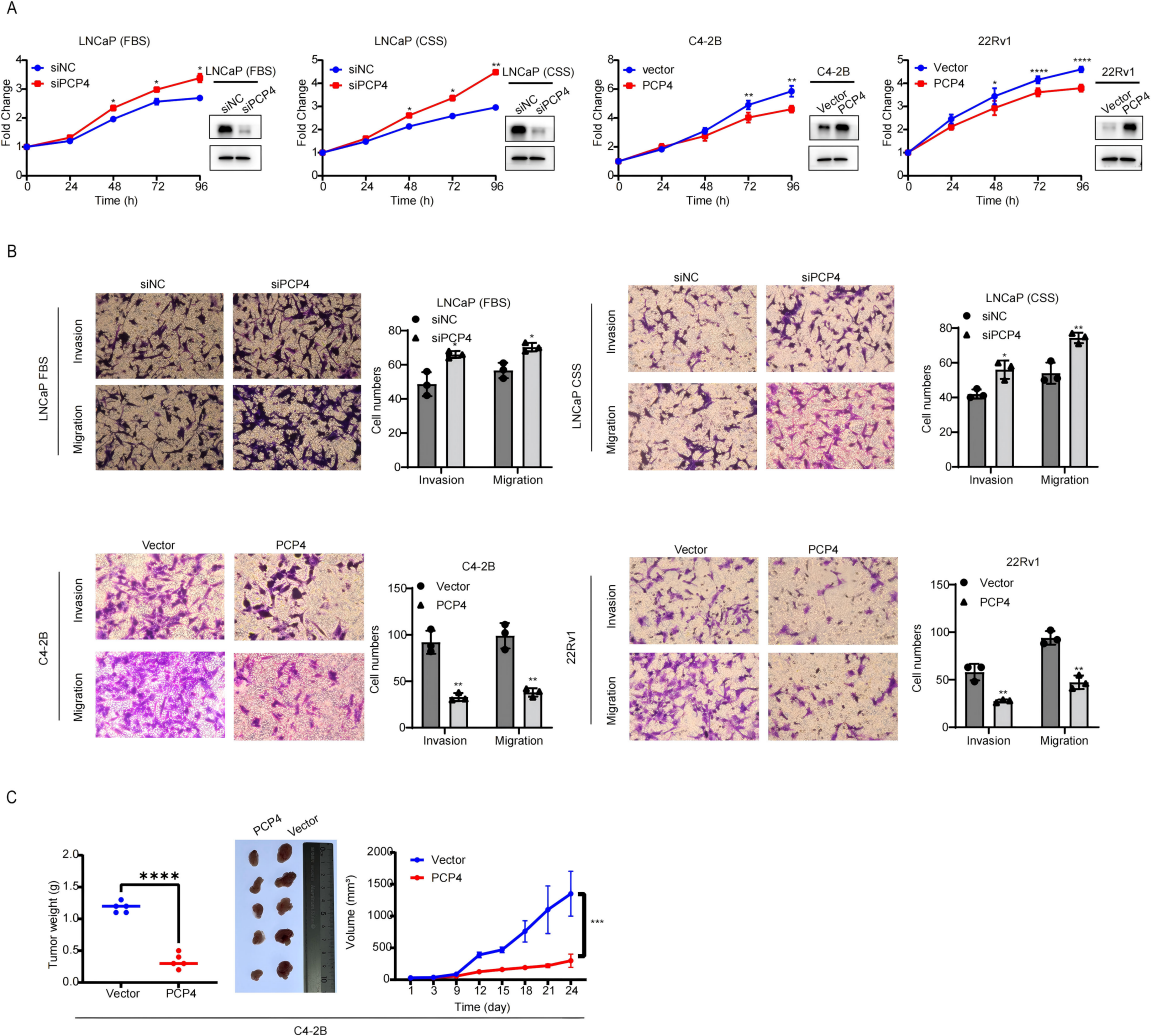
PCP4 inhibits PCa cell growth *in vitro* and *in vivo*. **(A)** Cell viability of PCa cell lines was assessed by MTS assays. The transfection efficiency was evaluated by Western blotting assay. **(B)** Trans-well assay was performed in PCa cell lines to assess the migration and invasion ability. **(C)** Xenograft PCa tumor growth upon PCP4 overexpression. C4-2B cells with stable overexpression of negative vector or PCP4 were injected subcutaneously into nude mice (5 mice per group). Tumor size was measured every 3 days. Data was shown as the means ± S.D, based on Student’s *t* test. ^*^ *p*<0.05, ^**^ *p*<0.01, ^***^ *p*<0.001, ^****^ *p*<0.0001.

RNA silencing was applied to deplete PCP4 expression in androgen dependent cell line LNCaP, while plasmid to overexpress PCP4 in CRPC cell line C4-2B and 22Rv1 (**Figure 3A**). Downregulation of PCP4 led to increased cell growth in LNCaP cells, regardless of whether androgen was depleted (using CSS) or not. Conversely, PCP4 overexpression decreased cell growth in C4-2B and 22Rv1 cells (**Figure 3A**). Downregulation of PCP4 also resulted in increased migration and invasion capabilities in LNCaP cells both in the presence and absence of androgen depletion. Similarly, PCP4 overexpression reduced cell growth in C4-2B and 22Rv1 cells (**Figure 3B**). Intriguingly, PCP4 knockdown significantly augmented the proliferation and invasion of LNCaP cells under androgen depletion compared to androgen-repletion conditions (using FBS).

Furthermore, PCP4 overexpression in C4-2B xenografts in castrated nude mice resulted in delayed tumor progression (**Figure 3C**). The mean tumor volume at 1 351 ± 353.0 mm (3) in C4-2B-Vector xenografts and 299 ± 104.0 mm (3) in C4-2B-PCP4 xenografts with tumor weight 1.180 ± 0.084 g and 0.340 ± 0.114 g, respectively.

### Subcellular location of PCP4 and PCP4 is androgen-responsive

**Figure 4:**
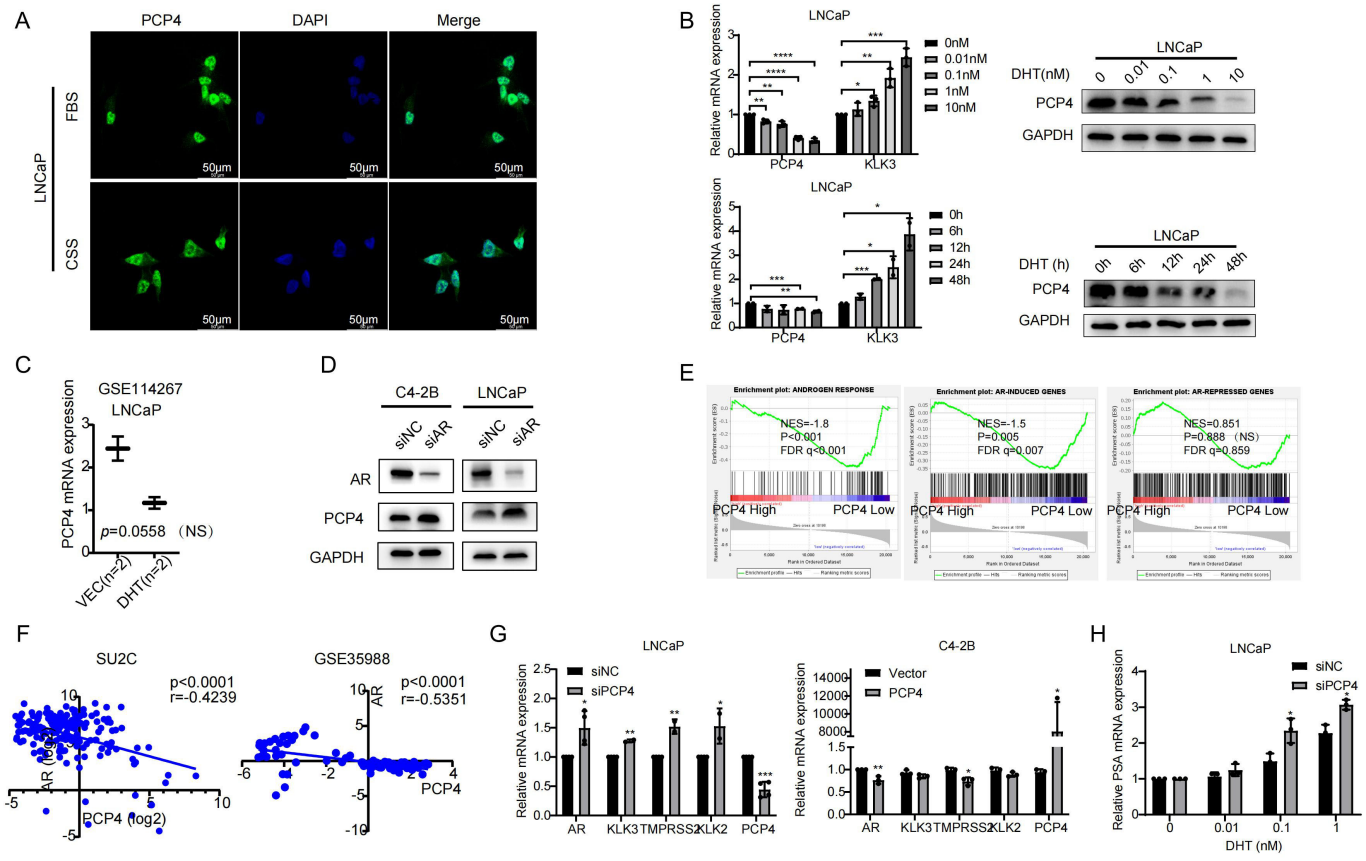
PCP4 is an AR-repressed gene and it regulates AR signaling in PCa cells. **(A)** PCP4 is distributed mainly in the cell nucleus and a small amount in the cytoplasm in LNCaP cells, regardless of whether androgen was depleted or not. FBS, fetal bovine serum. CSS, charcoal stripped fetal bovine serum. **(B)** Androgen responsiveness of PCP4 in PCa cell lines. After LNCaP cells treated by DHT with 0–10 nM for 24 hours or 1 nM for 0–48 hours, the mRNA and protein expression levels of PCP4 were measured by qRT-PCR and Western blotting assays. KLK3 was measured as a positive control. **(C)** Data was obtained from GEO dataset GSE114267. LNCaP cells were treated with negative control (Vec) or DHT. Expression profiling was measured by high throughput sequencing. NS, non-significant. **(D)** Western blotting of AR and PCP4 in PCa cells that were treated with AR siRNA. siNC, small interfering Negative Control. **(E)** Enrichment of AR-mediated gene program analyzed by GSEA based on TCGA database. TCGA samples were divided into 2 groups according to PCP4 mRNA expression level (High 25% vs. low 75%). GSEA was carried out to examine the enrichment of androgen-induced and androgen-repressed gene sets. ES, enrichment score. NS, non-significant. **(F)** Pearson correlation of mRNA expression between PCP4 and AR. Transcriptomic data from SU2C and GSE35988 clinical PCa cohort was used to perform correlation analysis. **(G)** Expression of AR and its target downstream genes in LNCaP and C4-2B cell lines which were pretreated with siRNA against PCP4 or negative control for 48 hours and quantified by qRT-PCR. **(H)** Real-time PCR measurement of endogenous KLK3 mRNA levels in LNCaP-siNC and LNCaP-siPCP4 cell lines treated with the indicated concentrations of dihydroxytestosterone. ^*^ *p*<0.05, ^**^ *p*<0.01, ^***^ *p*<0.001, ^****^ *p*<0.0001.

PCP4 protein is distributed mainly in the cell nucleus and a small amount in the cytoplasm in LNCaP cells, regardless of whether androgen was depleted (using CSS) or not (**Figure 4A**). The androgen-AR signaling plays a pivotal role in prostate tumorigenesis and progression, especially in the growth of CRPC. Given that PCP4 expression is most prominent in AR-positive LNCaP cells and AR serves as the primary target of DHT action in LNCaP cells (32), we utilized LNCaP cell line to identify whether PCP4 responds to androgen. Prostate specific antigen (PSA), an endogenous AR downstream target, was utilized as a positive control. LNCaP cells were first cultured in CSS medium for 3 days and then exposed to 0–10 nM DHT for 24 hours or 1 nM DHT for 0–48 hours. As depicted in **Figure 4B**, upon treatment with DHT, PCP4 decreased while PSA increased in a dose- and time-dependent manner at both mRNA and protein levels in LNCaP cells. The public dataset from GSE114267 also showed that PCP4 expression decreased when LNCaP was treated with DHT, even though the data was not significant which might be due to small sample size (**Figure 4C**). Conversely, down-regulation of AR in LNCaP and C4-2B cell lines led to PCP4 overexpression in both these two cell lines (**Figure 4D**). Based on these results, we infer that PCP4 is a novel DHT-repressed AR-target gene in PCa cells.

### PCP4 regulates AR signaling in PCa cells

Multiple public datasets were utilized to elucidate how PCP4 regulates AR signaling. GSEA analysis based on TCGA dataset demonstrated that AR responded gene sets were significantly enriched in PCP4-depleted samples (NES = -1.86; *p* < 0.001; FDR q < 0.001) and AR-induced genes were also enriched in PCP4 depleted samples (NES = -1.50; *p* = 0.005; FDR q = 0.007) (**Figure 4E**). Meanwhile, PCP4 mRNA expression was negatively correlated with AR expression in several public datasets, such as SU2C and GSE35988 (**Figure 4F**).

To explore the mechanisms of how PCP4 regulates AR signaling, we detected key molecules in AR signaling pathways, which were reported as downstream effectors of AR, including kallikrein related peptidase 2 (KLK3), TMPRSS2, kallikrein related peptidase 2 (KLK2) (**Figure 4G**). Down regulation of PCP4 in LNCaP cells increased the mRNA levels of AR, KLK3, TMPRSS2, KLK2. While PCP4 overexpression in C4-2B reduced the mRNA levels of AR and TMPRSS2. These results suggested that AR signaling was negatively related with PCP4 expression in PCa. Interestingly, qRT-PCR results showed that PCP4 downregulation enhanced the expression of the AR-regulated gene KLK3 in a dose-dependent manner (**Figure 4H**).

### PCP4 negatively regulates Ca^2+^/CAMKK2/AR signaling

**Figure 5:**
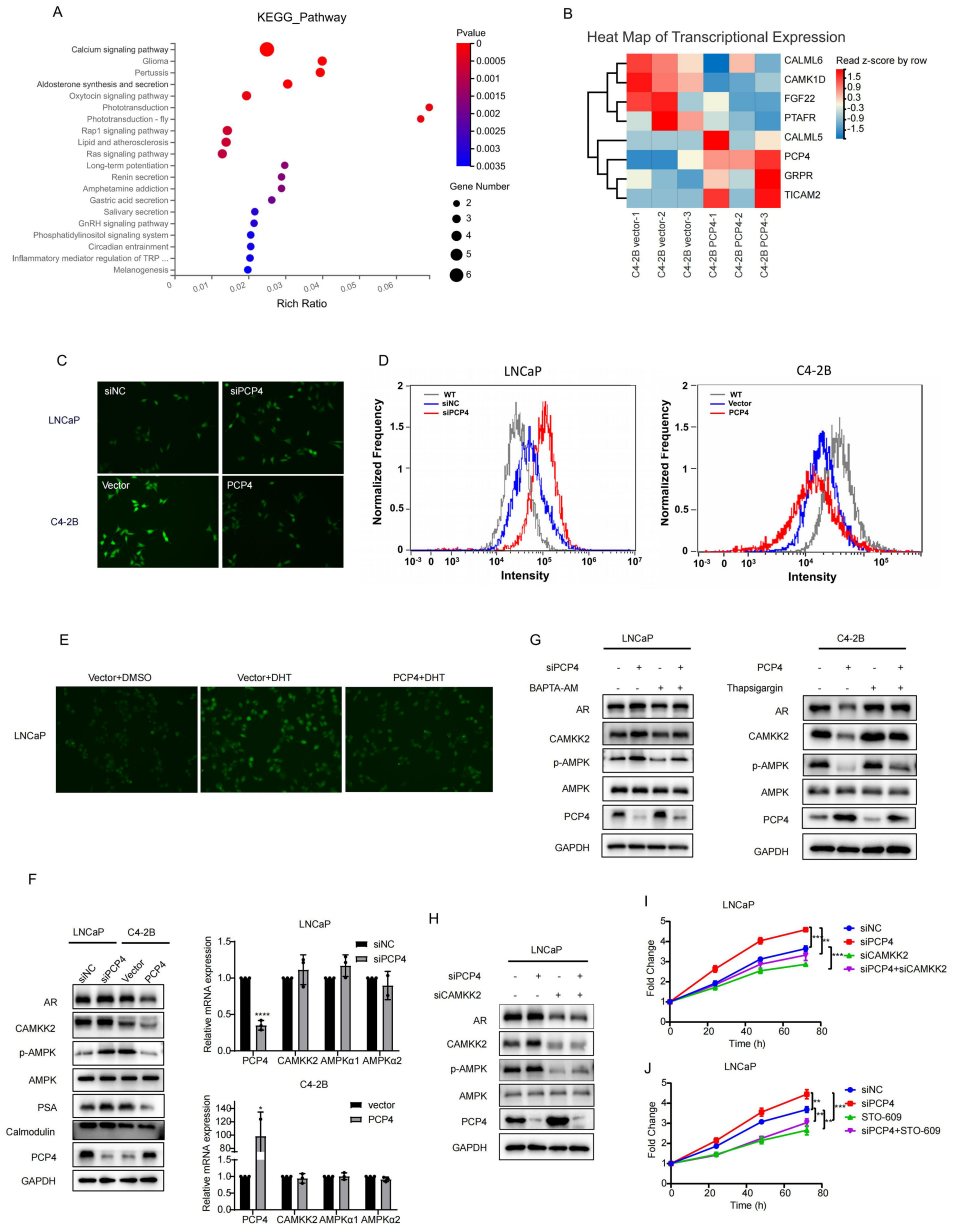
PCP4 negatively regulates Ca^2+^/CAMKK2/AR signaling. **(A)** Top 20 KEGG gene sets enriched in C4-2B cells transfected with PCP4 plasmids compared with negative vectors (Log FC>1, P<0.05). **(B)** Heat map of mRNA expression levels of the differentially expressed genes associated with Calium signaling pathway. **(C, D)** Intracellular Ca^2+^ levels assessed by inverted fluorescence microscope and flow cytometry after staining with Fluorescent prob Fluo-4 AM in LNCaP and C4-2B cells. **(E)** Intracellular Ca^2+^ levels assessed by inverted fluorescence microscope after staining with Fluorescent prob Fluo-4 AM in LNCaP cells with different treatment as indicated. **(F)** Western blot and q-RT PCR analyses of AR, CAMKK2, phosphorylated AMPK (p-AMPK), AMPK, PSA, Calmodulin and PCP4 in LNCaP and C4-2B with different treatments as indicated. **(G)** Western blotting of indicated proteins in cells treated with an inhibitor of microsomal Ca^2+^-ATPase Thapsigargin (1 μM, 6h) and Ca^2+^ chelator BAPTA-AM (10μM, 12h) respectively. **(H)** Western blotting results of AR, CAMKK2, phosphorylated AMPK (p-AMPK), AMPK and PCP4 in LNCaP and C4-2B cells. **(I-J)** MTS results of LNCaP cells with different treatments as indicated. The concentration of STO-609 was 10μM. h, hours. ^*^ *p*<0.05, ^**^ *p*<0.01, ^***^ *p*<0.001, ^****^ *p*<0.0001.

Then we carried out RNA-sequencing to investigate the mechanisms through PCP4 suppresses the aggressive phenotype of PCa cells. C4-2B cells were transfected with either negative vectors or PCP4 plasmids for 48 hours. After that, total RNA was extracted for RNA sequencing analysis. KEGG analysis showed that the dysregulated genes induced by PCP4 overexpression were mostly enriched in *Calcium signaling pathway*, which was consistent with its molecular function reported before (**Figure 5A**). The relative expression levels of genes associated with *Calcium signaling pathway* were showed in **Figure 5B**.

Intracellular Ca^2+^ acts as one of the most widespread and crucial second messenger molecules. The Ca^2+^ fluorescence images and flow cytometry results showed that intracellular Ca^2+^ concentration decreased remarkable in C4-2B cells after PCP4 overexpression compared to the control while elevated in LNCaP cells after PCP4 downregulation (**Figures 5C, D**).

Previous study indicated that DHT could accelerate Ca^2+^ influx and increase the Ca^2+^ concentration in PCa cancer cells (33). In this study, we observed that DHT elevated Ca^2+^ concentration was partially reversed by PCP4 overexpression (**Figure 5E**). These results indicated that DHT induced PCP4 downregulation was involved in the process of DHT induced Ca^2+^ influx.

Among all the molecules involved in the *Calcium signaling pathway*, the pro-tumorigenic role of CAMKK2 in prostate cancer was widely established. Previous evidence has demonstrated that CAMKK2 expression was induced by AR and it in turn stabilized AR to promote its transcriptional activity and cell cycle progression. This interaction forms a novel positive feedback loop within the PCa (20, 21). Consequently, we hypothesized that the mechanism of PCP4 regulating PCa progression might be linked to Ca^2+^/CAMKK2/AR signaling.

CAMKK2, a crucial factor in the progression of PCa and transition to CRPC, has mostly been linked to phosphorylation of AMPK at threonine 172 (AMPK(Thr172)) in an AR-dependent manner (19). To examine whether PCP4 regulates CAMKK2 signaling pathway, AMPK and p-AMPK were tested as downstream effectors of CAMKK2. PCP4 did not induce remarkable changes of CAMKK2 in mRNA level. However, downregulation of PCP4 in LNCaP cells increased the protein levels of both CAMKK2 and p-AMPK. Conversely, the overexpression of PCP4 in C4-2B cells reduced CAMKK2 and p-AMPK protein expression levels (**Figure 5F**).

Additionally, treatment with Ca^2+^-chelator BAPTA-AM effectively reversed the PCP4-mediated inhibition of CAMKK2; Conversely, the CAMKK2-activating effect induced by PCP4 knockdown was significantly reversed by thapsigargin (a Ca^2+^-ATPase inhibitor) (**Figure 5G**). Critically, CAMKK2 depletion reversed PCP4 depletion-induced activation of p-AMPK and cell proliferation in LNCaP cells (**Figure 5H**). STO-609 is a regular inhibitor of CAMKK2 (34). The MTS results showed that whether it was siRNA or STO-609, both reversed PCP4 depletion-induced cell proliferation in LNCaP cells (**Figures 5I, J**). These findings collectively suggested that the downregulation of PCP4 promoted PCa progression by activating Ca^2+^/CAMKK2/AMPK/AR signaling axis.

### PCP4 promotes degradation of CAMKK2 in PCa cells

**Figure 6:**
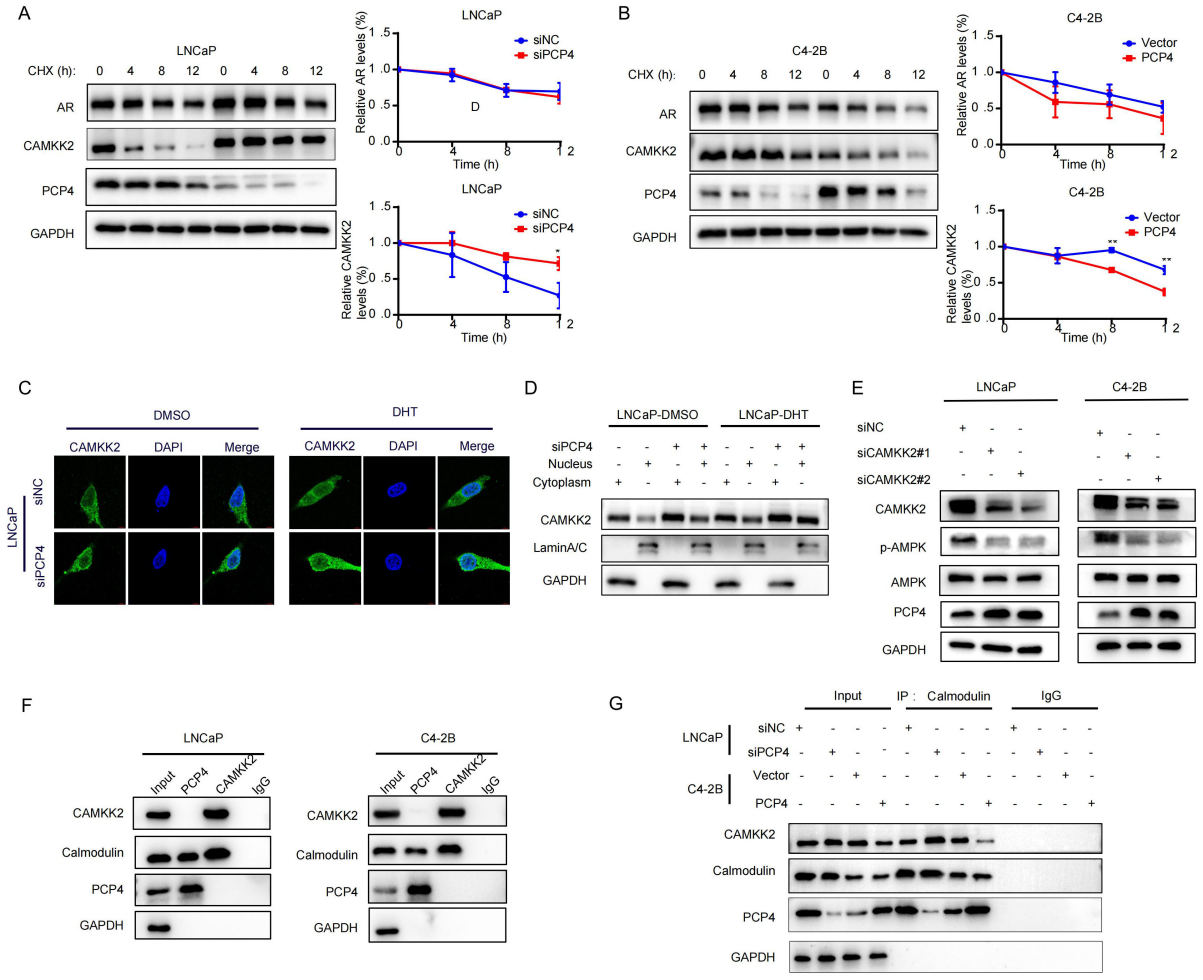
The loop between PCP4 and CAMKK2. **(A, B)** AR and CAMKK2 protein levels by western-blotting. LNCaP cells were transfected with siPCP4 while C4-2B cells were transfected with PCP4 plasmids. h, hours. **(C, D)** Subcellular location of CAMKK2 proteins in LNCaP cells by immunofluorescence and subcellular fractionation as indicated. **(E)** The protein levels of PCP4, AMPK and p-AMPK in LNCaP and C4-2B cells transfected with siCAMKK2. **(F, G)** The binding potential between CaM and PCP4 or CAMKK2 was performed in the total lysates by Co-IP assays in LNCaP and C4-2B cells. IgG was used as negative control. GAPDH served as a loading control. ^*^ *p*<0.05, ^**^ *p*<0.01.

Since PCP4 modulates CAMKK2 post-transcriptionally without altering its transcriptional expression, we next investigated whether PCP4 affects CAMKK2 or AR protein degradation in PCa cells. LNCaP and C4-2B cell lines were treated with cycloheximide (CHX) to block *de novo* protein synthesis. In LNCaP cells, downregulation of PCP4 significantly decelerated the degradation of CAMKK2 in LNCaP cells, while PCP4 overexpression remarkably accelerated the degradation of CAMKK2 in C4-2B cells (**Figures 6A, B**). These results indicated that PCP4 could promote the degradation of CAMKK2 protein.

### Subcellular localization of CAMKK2

Previous study suggested that CAMKK2 staining intensity increased with the Gleason score of tumors and the staining pattern shifted from predominantly cytoplasmic to perinuclear and nuclear (21). To explore whether PCP4 influences the nuclear translocation of CAMKK2, we treated with DHT or vehicle, then tested CAMKK2 localization by subcellular fractionation and immunofluorescence microscopy in PCP4 silenced LNCaP cells compared with control. The results showed that intracellular CAMKK2 was increased when PCP4 was downregulated whether LNCaP cells were treated with DHT or DMSO. Compared with DMSO group, CAMKK2 was increased after DHT treatment, and downregulation of PCP4 enhanced the CAMKK2 upregulation induced by DHT treatment. The increasement of CAMKK2 were both observed in both cytoplasmic and nuclear compartments (**Figures 6C, D**).

### CAMKK2 negatively regulates PCP4 expression

We noticed that downregulation of CAMKK2 increased the PCP4 protein expression level in LNCaP cells (**Figure 5H**). To confirm this finding, downregulation of CAMKK2 using siRNA was carried out in both LNCaP and C4-2B cell lines and the results verified this finding (**Figure 6E**). This suggests that CAMKK2 might form a feedback loop with PCP4.

### PCP4 regulates CAMKK2 binding with CaM

Co-IP results showed that both PCP4 and CAMKK2 protein could bind with CaM, while PCP4 did not bind with CAMKK2 (**Figure 6F**). The binding between CAMKK2 and CaM increased when PCP4 was downregulated in LNCaP and decreased when PCP4 was overexpressed in C4-2B, which might a result of the difference in CAMKK2 protein level (**Figure 6G**).

## Discussion

**Figure 7:**
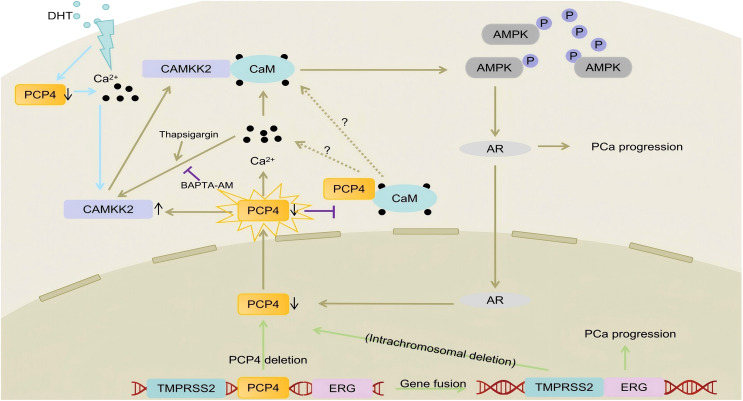
The mechanisms of PCP4 deletion/downregulation promote PCa progression. PCP4 gene deletion leads to the downregulation of PCP4 expression. PCP4 could regulate the intracellular Ca^2+^ concentration. Decreased PCP4 expression leads to the increased intracellular Ca^2+^ concentration and CAMKK2 protein expression. In that, the binding of CAMKK2 and CaM is also increased and the CAMKK2/AMPK/AR signaling is activated. The increased AR signaling in turn decreases PCP4 expression, forming a positive feedback loop and then leads to PCa progression. Interestingly, the using of Thapsigargin or BAPTA-AM could partly reverse the influence of PCP4 on CAMKK2, indicating that PCP4 could regulate CAMKK2 expression not only by modulating the stabilization of protein but also through Ca^2+^ signaling.

PCa represents the most prevalent malignancy of the prostatic epithelium (1). Although androgen deprivation therapy (ADT) is initially effective in managing PCa by reducing androgen levels that drive cancer growth, resistance often develops over time, leading to the progression of CRPC, a more advanced and aggressive stage of PCa (35). This study reveals that the downregulation of PCP4, potentially due to *PCP4* gene deletion, drives the progression of PCa, particularly CRPC, through modulation of Ca^2+^/CaM/CAMKK2/AMPK/AR signaling axis (**Figure 7**).

The *TMPRSS2-ERG* gene fusion, a hallmark of PCa, arises via intrachromosomal deletion (50% cases) or insertional rearrangement. Notably, deletion-derived fusions correlated with aggressive tumor behavior (3), potentially due to concurrent loss of interstitial genes (e.g., *FAM3B*) that modulate prognosis (4). Strikingly, *PCP4* resides within the *TMPRSS2-ERG* interstitial region. Public database analyses revealed that PCP4 is downregulated in CRPC and inversely correlated with advanced clinicopathological features (e.g., higher Gleason scores, pTNM stages) and poor survival. While co-deletion of *PCP4* and *TMPRSS2-ERG* fusion events has observed, RNA-sequencing highlighted PCP4’s role in Ca^2+^-dependent pathways, prompting further investigation into whether *PCP4* loss synergizes with *TMPRSS2-ERG* fusion as a “second hit” or independently drives CRPC progression. In order to better understand it, we conducted a more comprehensive analysis using public databases TCGA and SU2C (**Supplementary Figure 1**). The results of prognostic analysis showed no significant difference between each group, regardless of *PCP4* gene deletion and the presence of *TMPRSS2-ERG* gene fusion. Public database analysis suggested that PCP4 gene deletion and *TMPRSS2-ERG* gene fusion might not have a clear cooperative relationship in the PCa progression. However, this needs to be further confirmed by additional basic experiments *in vitro* such as knockdown of PCP4 in ERG-overexpressing cell models or *in vivo* experiments.

AR, a ligand-dependent nuclear transcription factor, binds to testosterone or DHT, driving the transcription of AR-responsive genes and promoting prostate cell proliferation and survival (36). The fusion of *TMPRSS2* and *ETS* genes, particularly involving *ERG*, regulates the AR pathway (37) and impairs tumor proliferation checkpoints (38). Our data identify *PCP4* as a novel DHT-repressed AR-target gene, with marked under expression in CRPC. Functionally, PCP4 overexpression suppressed PCa cell and cell-line-derived xenograft tumor growth, whereas PCP4 knockdown exacerbated proliferation and invasion under androgen-depleted conditions compared to adrogen-repletion conditions. Notably, PCP4 downregulation amplified AR-driven KLK3 expression dose-dependently, positioning PCP4 as a novel therapeutic target in CRPC.

RNA-sequencing of PCP4-overexpressing C4-2B cells revealed enrichment in *calcium ion binding* pathways, aligning with PCP4’s known interaction with CaM (9). Intracellular Ca^2+^, a key second messenger, regulates proliferation and transcription via effectors like CAMKK2, a Ca^2+^/CaM-dependent kinase overactive in PCa (39). DHT elevated cytosolic Ca^2+^ and suppressed PCP4 expression, while PCP4 overexpression attenuated DHT-induced Ca^2+^ influx, suggesting PCP4 was involved in the DHT induced Ca^2+^ influx. Mechanistically, PCP4 downregulation reduced CaM binding, elevating cytosolic Ca^2+^ levels. This, in turn, increased CAMKK2 protein stability and enhanced CaM-CAMKK2 interaction. CAMKK2 stabilization activates AMPK, which stabilizes AR via p38 signaling (40). Paradoxically, while CAMKK2/AMPK signaling promotes AR activity, PCP4 downregulation did not directly stabilize AR, implication CAMKK2 as the primary effector. In our current study, we observed that upon PCP4 overexpression or downregulation, it was the total protein expression of CAMKK2 that underwent alterations. Therefore, we refrained from using the p-CAMKK2(Ser511) as an indicator of enhanced CAMKK2 activity (40), which might be controversial as prior studies have demonstrated (41), or just merely reflects an increase in the overall level of CAMKK2 protein. To date, the pro-tumorigenic activity of CAMKK2 in PCa has mostly been linked to the downstream factor AMPK, so we used p-AMPK and AMPK as the markers of CAMKK2 activity.

As the results of this study showed, PCP4 expression was influenced by different mechanisms, including gene loss, androgen signaling regulation, and CAMKK2-mediated inhibition. The deletion of the PCP4 gene is a molecular event that affects the expression of PCP4 at the transcriptional level. The focus of this study is to discover that the deletion or low expression of PCP4 affects the CAMKK2-AR signaling pathway. Additionally, at the phenotypic level, it was found that this signaling pathway further reduces the expression of PCP4 through a positive-feedback mechanism. We only observed the results at the phenotypic level. There has been no further exploration of the specific mechanisms, which we suspect may be related to transcription due to AR plays the role of transcription factors in most situations. As for which of these three events or molecules is more crucial for PCP4, we assume that it ought to be deliberated upon in diverse situations. This is also related to cell models or the specimen tissues themselves. Also, in most cases, we believe that they probably shouldn’t be compared in terms of which one is more important, but rather they jointly complete this influence on PCP4.

The progression of PCa, particularly CRPC is complex and involves more than genetic and epigenic mechanisms. Consequently, It is reasonable to believe that combinatorial therapies co-targeting oncogenic pathways would provide optimal clinical benefits. Evidence in this study demonstrates that the CAMKK2 inhibitor, STO-609, reversed PCP4 depletion-induced cell proliferation in LNCaP cells. These results highlight the therapeutic potential of CAMKK2 inhibitors for clinical use, especially in patients with PCP4 deletion.

While the relationship between the *TMPRSS2-ERG* fusion and *PCP4* deletion or CAMKK2 activity was not specifically investigated here, no evidence emerged to suggest CAMKK2 inhibitors affect tumor progression in *TMPRSS2-ERG* gene fusion cases. Currently, CAMKK2 inhibitor are not used clinically, and rigorous clinical trials, including studies in mouse models and humans, remain essential to evaluate their safety and efficacy before clinical application. Nevertheless, this study supports the strategy of identifying key driver genes within specific patient subgroups (e.g., those with *PCP4* deletion) and designing effective inhibitors to block their signaling pathways. This precision medicine approach holds promise for improving patient outcomes.

This study establishes PCP4 downregulation as a pivotal event in PCa progression, especially the CRPC progression, disrupting Ca^2+^ homeostasis to amplify CAMKK2/AMPK/AR signaling. Pharmacological modulation of Ca^2+^ (via thapsigargin/BAPTA-AM) confirmed this pathway, yet key questions remain: 1. Does competitive binding between PCP4 and CAMKK2 for CaM drive Ca^2+^ dysregulation? 2. How does PCP4 regulate CAMKK2 protein stability? By identifying PCP4 as a gatekeeper of Ca^2+^/CAMKK2 signaling, this work opens avenues for targeting this axis in CRPC, particularly in tumors with PCP4 deletion or downregulation.

## Conclusion

This study reveals that PCP4 has the potential to inhibit PCa progression. The downregulation of PCP4 could enhance the Ca^2+^/CAMKK2/AMPK/AR pathway. These findings provide an attractive target for PCa progression.

## Data Availability

The datasets presented in this study can be found in online repositories. The names of the repository/repositories and accession number(s) can be found below: https://www.ncbi.nlm.nih.gov/, GSE293745.
