# Supplementary figures and images for: PCP4 inhibits the progression of prostate cancer through Ca2+/CAMKK2/AMPK/AR pathway
Source: Front Immunol. 2025 Jul 17;16:1616046. doi: 10.3389/fimmu.2025.1616046 (PMC12311239; doi:10.3389/fimmu.2025.1616046)

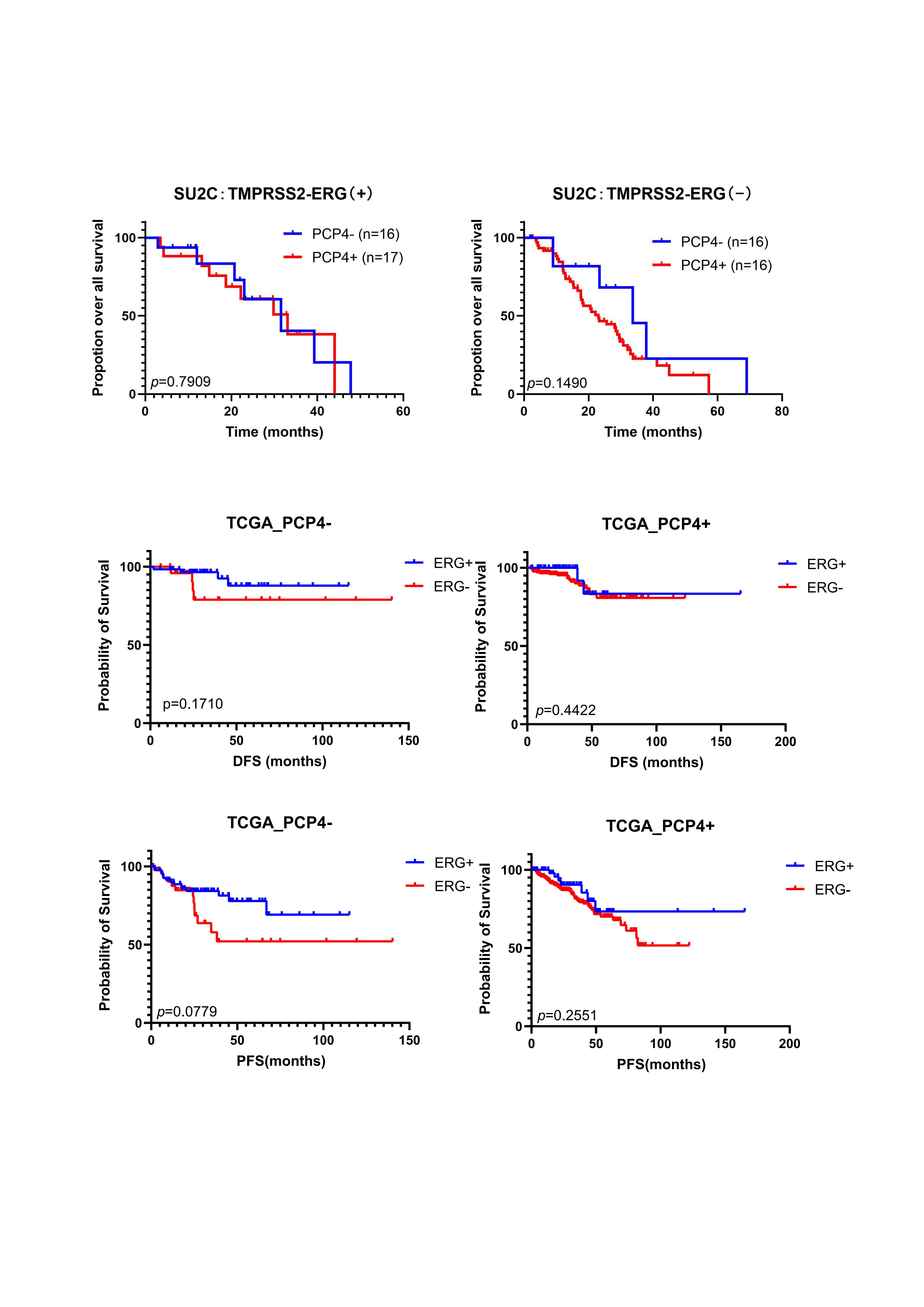

Supplement: Supplementary file 1 [file Image1.jpeg]
